# *Escherichia coli* ST155 as a production-host of three different polyvalent phages and their characterisation with a prospect for wastewater disinfection

**DOI:** 10.1038/s41598-022-24134-4

**Published:** 2022-11-12

**Authors:** Amrita Salim, Ajith Madhavan, Suja Subhash, Megha Prasad, Bipin G. Nair, Sanjay Pal

**Affiliations:** School of Biotechnology, Amrita Vishwa Vidyapeetham, Kollam, Kerala 690525 India

**Keywords:** Biotechnology, Microbiology, Environmental sciences

## Abstract

Bacteriophages are generally specific, and a cocktail of phages is needed to combat different bacterial targets. Their production usually requires pathogenic isolation hosts. We identified a novel strain, *Escherichia coli* ST155, that could serve as a production host for three different polyvalent phages (ϕPh_SE03, ϕPh_SD01, and ϕPh_EC01), thus superseding the use of individual isolation hosts. Upon propagation in *E. coli* ST155, the phages demonstrated differential intergeneric infectivity against *Salmonella enterica*, *E. coli* OP50*, Shigella dysenteriae*, *E. coli* MDR*,* and *Acinetobacter baumannii*. Phages were characterised based on morphology, latent period, burst size, the efficiency of plating, and restriction enzyme profile. Survival assay on *Caenorhabditis elegans,* the absence of Shiga toxin, and enterotoxigenic *E. coli* virulence genes indicated that *E. coli* ST155 could be non-pathogenic. Lack of antibiotic resistance and absence of functional prophages rendered the host suitable for environmental applications. As a proof-of-concept, phage ϕPh_SE03 was produced in ST155 by employing a unique Bacteriophage Amplification Reactor-Lytics Broadcasting System and was simultaneously disseminated into *S. enterica* augmented wastewater, which resulted in a 3-log reduction in 24 h. The study establishes the potential of *E. coli* ST155 as a phage production host thereby minimising the possibility of accidental release of pathogenic hosts into wastewater.

## Introduction

The use of bacteriophages as an alternative solution for wastewater disinfection and malodour mitigation is gaining extensive attention amongst biologists and engineers alike. Two important reasons are host specificity and non-toxicity. In comparison, chemical disinfectants generate carcinogenic by-products and cause adverse and detrimental effects on beneficial organisms involved in the remediation of wastewater^1–5^. Moreover, phages can help reduce the burden of antimicrobial resistance (AMR) by preventing the propagation of AMR pathogens in wastewater, which otherwise serves as the breeding ground and source of infection in humans and animals^6,7^. Phages can also find applications in addressing several other problems associated with wastewater, namely foaming of activated sludge, sludge dewaterability and digestibility^8–11^.

However, translation into technology would be replete with myriad challenges, most notably the use of pathogenic hosts in propagating phages. The presence of non-pathogenic, high-efficiency production hosts would go a long way in the widespread application of phages in wastewater settings. Ideal features of a production host include the ability to host different phages, non-pathogenicity and the ability to generate a high number of progeny phages. Wastewater applications also demand a device to propagate and disseminate phages with safety provisions to prevent the inadvertent introduction of pathogenic bacterial hosts. Bacteriophage Amplification Reactor-Lytics Broadcasting System (BAR-LBS) is a membrane-based device developed by the authors to disseminate phages in wastewater^12^ selectively. BAR-LBS, in conjunction with a production host, can effectively subvert the issue of the unintentional release of pathogenic hosts into the environment^12–14^. Moreover, in a heterogeneous system such as wastewater, bacteriophage-based applications face the challenge of the need to deal with taxonomically distant bacteria that are implicated in the infection and biogenesis of malodour. The prevalence of polyvalent phages in wastewater reiterates the importance of operating disseminating systems with polyvalent phages targeting various bacterial species and strains^15,16^. Additionally, introducing a production host that can propagate multiple phages of polyvalent nature can pave the way for mobilising the application of phages^6^ as a practical solution, primarily in decentralised sanitation systems.

A non-pathogenic production host, *Escherichia coli* ST155, was used to proliferate three polyvalent phages—ϕPh_SE03, ϕPh_SD01, and ϕPh_EC01. The phages were characterised following propagation in the production host in terms of morphology, latent period, burst size, the efficiency of plating (EOP), and restriction digestion profile. The non-pathogenic nature of *E. coli* ST155 was validated through the analysis of its whole genome sequence and through survival assay in the *Caenorhabditis elegans* model. The antibiotic resistance profile and presence of prophages in the genome were also determined. In establishing the proof-of-concept, the production host was used to produce ϕPh_SE03 in the BAR-LBS which in turn disseminated the phages into *Salmonella enterica* augmented wastewater to study the effectiveness of phages in mitigating pathogenic bacteria. This production host, combined with a simple phage dissemination device such as BAR-LBS, gives good prospects for facile and more targeted application of phages to wastewater treatment.

## Results

### Characterisation of phage isolates

#### Morphology

**Figure 1:**
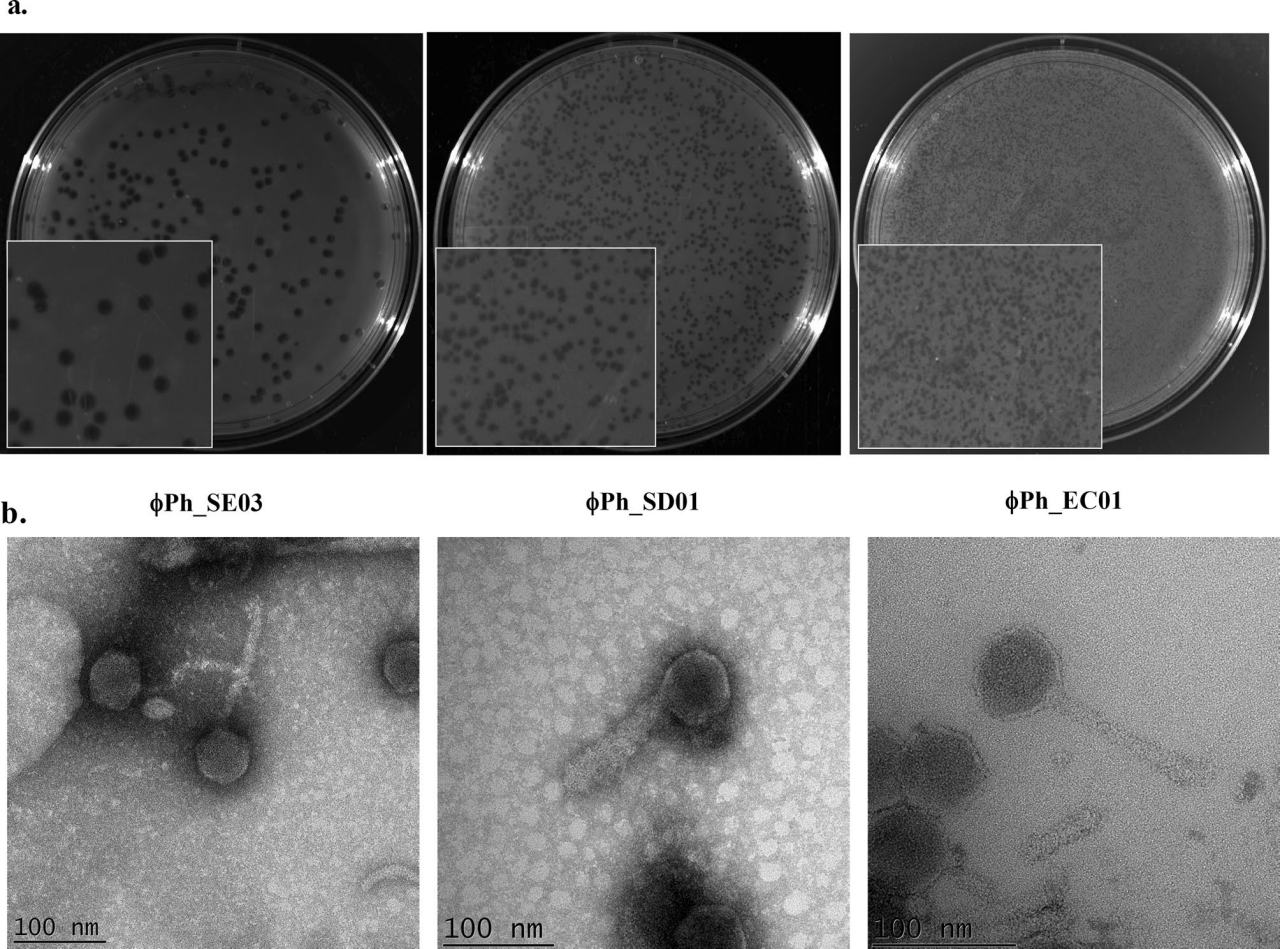
Morphological characterisation of phages. (**a**) Plaque morphology of phages ϕPh_SE03, ϕPh_SD01 and ϕPh_EC01 formed against *E. coli* ST155 determined by double agar overlay assay. (**b**) TEM morphology of the negatively stained phages ϕPh_SE03, ϕPh_SD01 and ϕPh_EC01 with their respective scale bars (100 nm).

All three phages propagated in *E. coli* ST155 maintained high titres (ϕPh_SE03—4 × 10^10^ PFU/mL, ϕPh_SD01—1.5 × 10^10^ PFU/mL, and ϕPh_EC01—1.3 × 10^10^ PFU/mL) and exhibited distinct plaque morphologies on double agar overlays, as shown in Fig. 1a. Phage ϕPh_SE03 showed large, clear and well-defined plaques; on the other hand, ϕPh_SD01 produced medium-sized plaques with frayed edges and ϕPh_EC01 in turn produced pinhead clear plaques. TEM analysis yielded broad morphological details along with its dimensions (*n* = 3). All isolated phages viz. ϕPh_SE03, ϕPh_SD01 and ϕPh_EC01 belonged to the class *Caudoviricetes* with typical icosahedral heads and are tailed (Fig. 1b). Phage ϕPh_SE03 has a capsid diameter of 58 nm and a tail length of 129 nm, while Phage ϕPh_SD01 has a capsid diameter of 66 nm and a tail length of 112 nm. Similarly, capsid diameter for the phage ϕPh_EC01 was 63 nm with a tail length of 116 nm.

#### Tropism of phages

**Figure 2:**
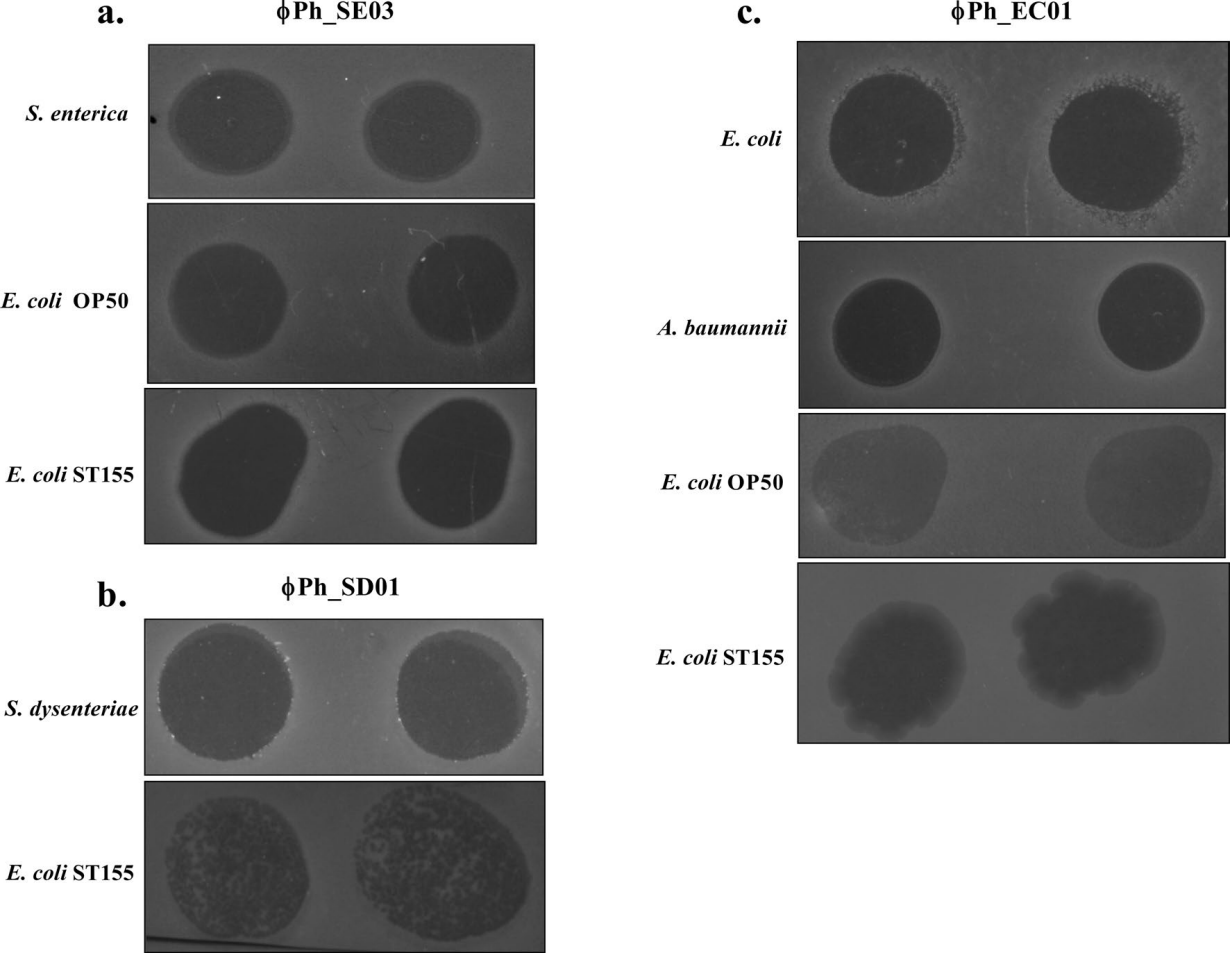
Host range of the three polyvalent phages propagated in *E. coli* ST155 determined by spot assay. (**a**) Clear lysis of *S. enterica, E. coli* OP50 and *E. coli* ST155 by phage ϕPh_SE03. (**b**) Phage ϕPh_SD01 shows a zone of lysis in *S. dysenteriae* and *E. coli* ST155 and (**c**) Phage ϕPh_EC01 shows lysis in *E. coli* MDR, *A. baumannii, E. coli* OP50 and *E. coli* ST155.

**Figure 3:**
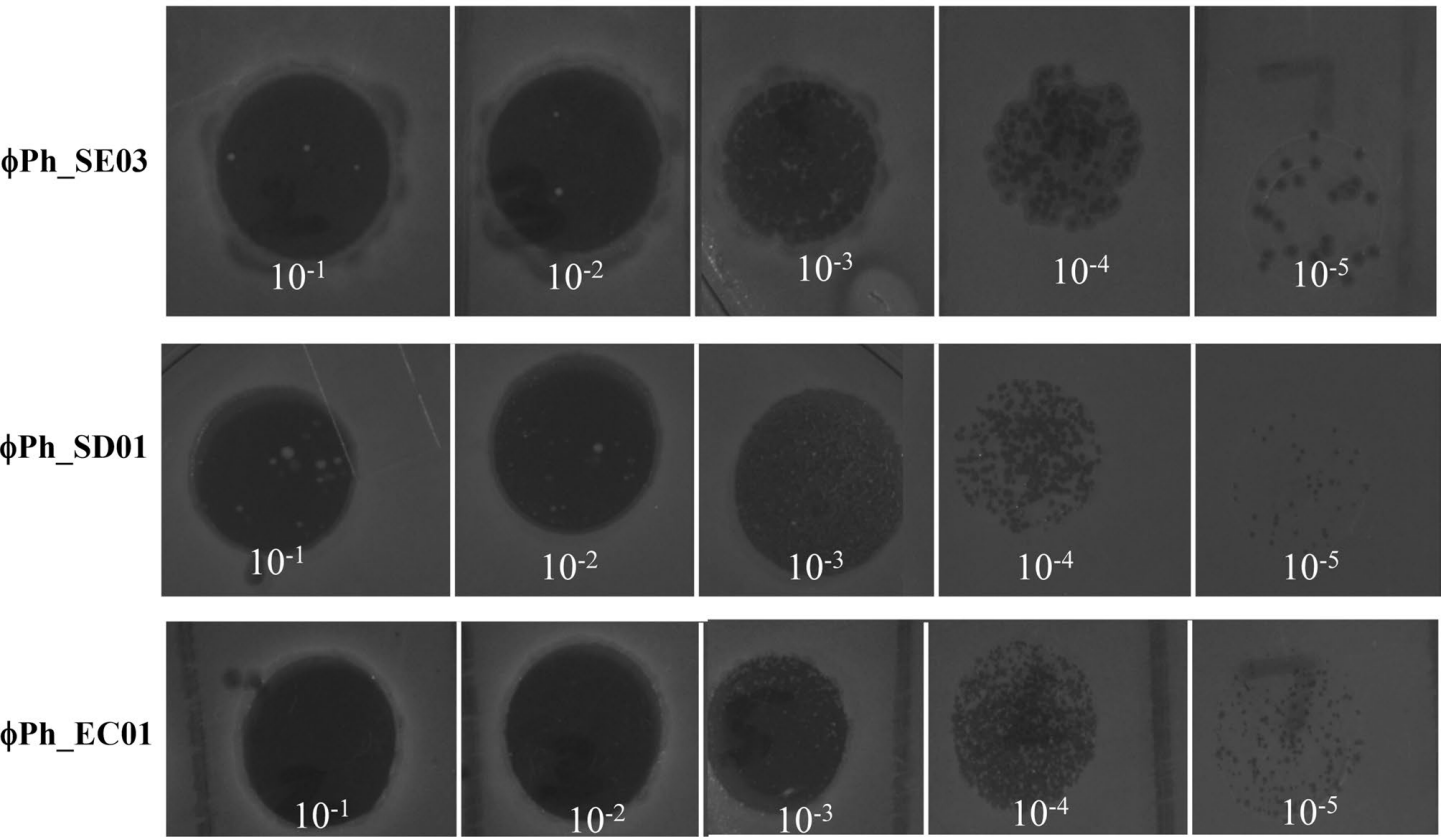
Spot assay of ϕPh_SE03, ϕPh_SD01 and ϕPh_EC01 against *E. coli* ST155 show characteristic plaques at higher dilutions confirming the presence of phage virions in the lysates.

The host ranges of the phages were tested against 16 different bacterial strains via spot assay following propagation in the production host. The zone of lysis indicated the host's susceptibility (Table 1). Phage ϕPh_SE03 could lyse *S. enterica* and *E. coli* OP50, while phage ϕPh_SD01 was active against *Shigella dysenteriae* (Fig. 2a,b). On the contrary, phage ϕPh_EC01 showed activity against *E. coli* MDR and other alternative hosts, namely *Acinetobacter baumannii* and *E. coli* OP50 (Fig. 2c). Bacteriocin’s involvement in generating clearing zones was negated by spotting serially diluted lysate. Lysate with phages produced characteristic plaques unlike bacteriocins at higher dilutions as shown in Fig. 3.

**Table 1:** Summary of the host range of polyvalent phages propagated in the production host *E. coli* ST155.

S. No	Bacteria	ϕPh_SE01	ϕPh_SD01	ϕPh_EC01
1	*E. coli* ST155	+	+	+
2	*E. coli* MDR	−	−	+
3	*E. coli* OP50	+	−	+
4	*S. enterica*	+	−	−
5	*S. dysenteriae*	−	+	−
6	*K. pneumoniae*	−	−	−
7	*K. quasipneumoniae*	−	−	−
8	*K. aerogenes*	−	−	−
9	*K. variicola*	−	−	−
10	*V. cholerae*	−	−	−
11	*S. marcescens*	−	−	−
12	*P. vulgaris*	−	−	−
13	*A. baumannii*	−	−	+
14	*P. aeruginosa* PAO1	−	−	−
15	*P. aeruginosa*	−	−	−
16	*S. aureus*	−	−	−
17	*L. fermentum*	−	−	−

#### Latent period and burst size

**Figure 4:**
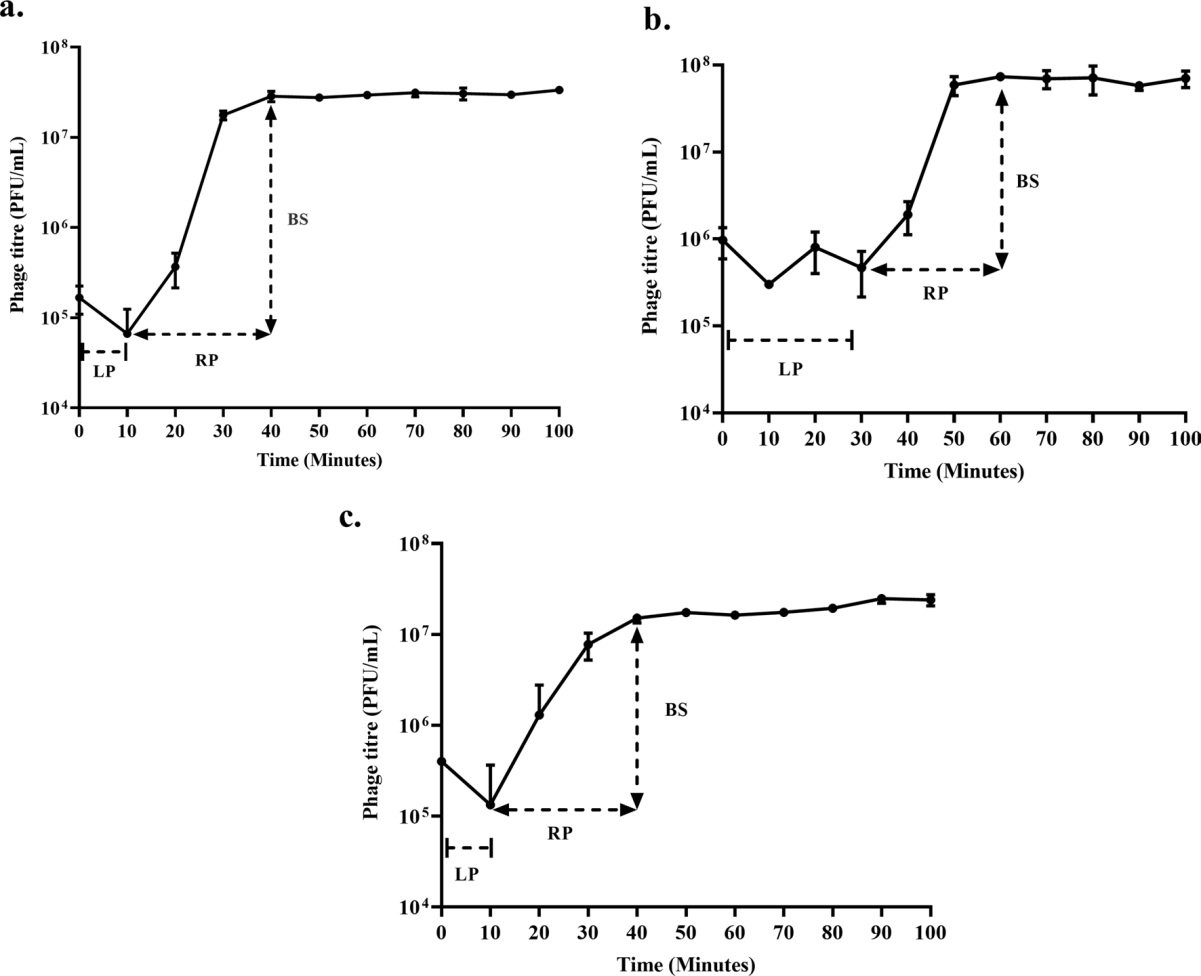
One-step growth curve. (**a**) ϕPh_SE03, (**b**) ϕPh_SD01, (**c**) ϕPh_EC01. The latent period (LP), rise period (RP) and burst size (BS) are indicated for all the phages for 100 min. The error bar represents the standard deviation from three replicates from independent experiments.

The phage infection parameters were determined with a one-step growth curve assay in *E. coli* ST155. The latent period of phage ϕPh_SE03 and ϕPh_EC01 was 10 min, while phage ϕPh_SD01 had an extended latent period that lasted up to 30 min. The burst sizes in phages ϕPh_SE03, ϕPh_SD01 and ϕPh_EC01 were 166 ± 10, 122 ± 31 and 44 ± 8.3 PFU/ infected cells, respectively (Fig. 4). The fact that *E. coli* ST155 is high yielding with regard to phage titre would augur well for its usage in BAR-LBS in disseminating high titre of phages.

#### High EOP of *E. coli* ST155

The EOP of the polyvalent phages were tested with the alternative hosts using phages propagated through the production host, as indicated in Table 2. The EOP of the production host was significantly higher than the rest of the other sensitive hosts, showing its potential for amplifying phages with a high titre, particularly during large-scale propagation.

**Table 2:** EOP of phages propagated in the production host *E. coli* ST155 with that of the alternative hosts using spot assay. SD represents the standard deviation of three replicates from independent experiments.

Phage	Host	Titre (PFU/mL)	EOP	SD
ϕPh_SE03	*S. enterica*	1.84E+07	8.40E−04	8.89E−04
	*E. coli* OP50	2.49E+07	1.18E−03	1.31E−03
	*E. coli* ST155	3.77E+10	1.00E+00	0.00E+00
ϕPh_SD01	*S. dysenteriae*	1.18E+10	3.35E−01	3.31E−01
	*E. coli* ST155	4.47E+10	1.00E+00	0.00E+00
ϕPh_EC01	*E. coli* MDR	5.76E+08	8.06E−02	8.82E−02
	*A. baumannii*	7.77E+06	9.79E−04	1.02E−03
	*E. coli* OP50	1.61E+05	2.17E−05	2.33E−05
	*E. coli* ST155	2.15E+10	1.00E+00	0.00E+00

#### Restriction digestion profile

**Figure 5:**
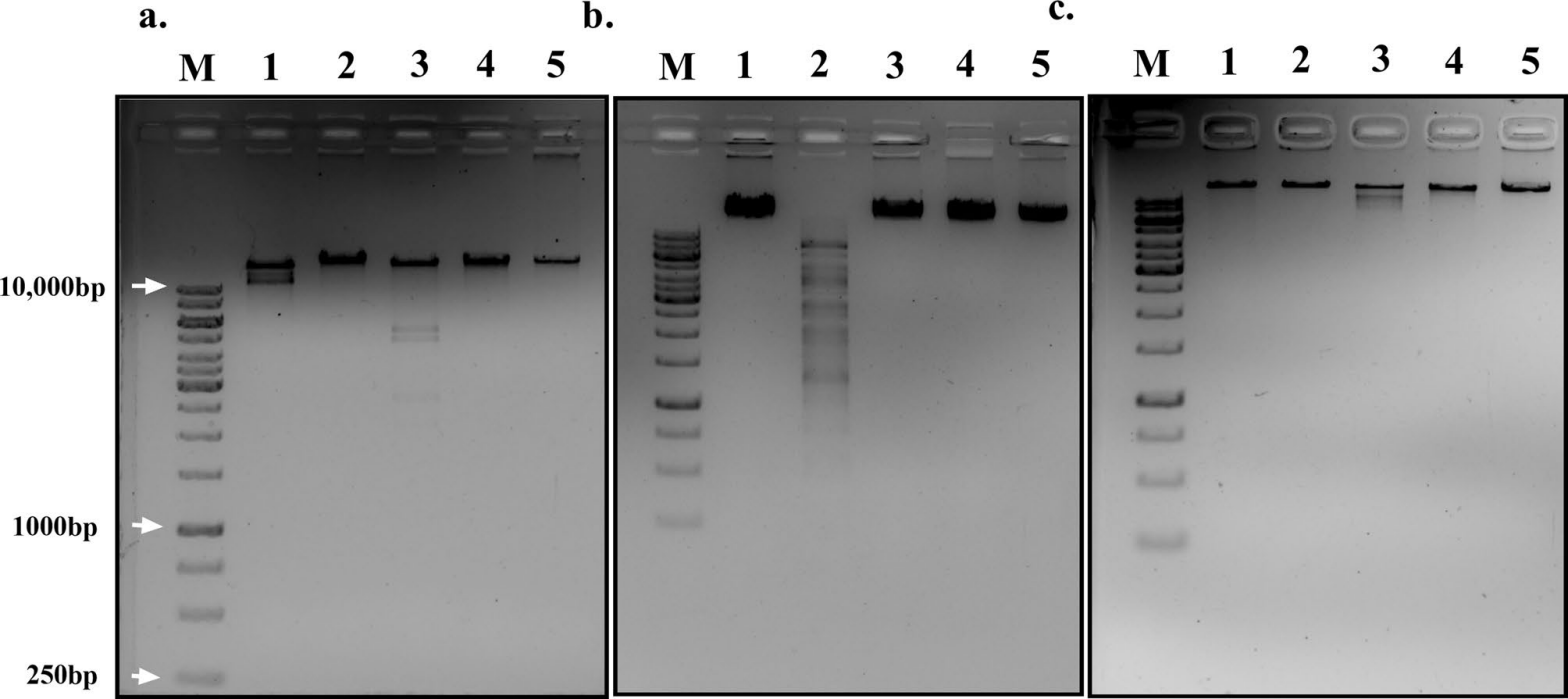
Restriction enzymes digested phage DNA electrophoresed on a 1% agarose gel stained with ethidium bromide (highlighted in the white box). (**a**) ϕPh_SE03, (**b**) ϕPh_SD01 and, (**c**) ϕPh_EC01. M – Marker 1 kb (GeneRuler, ThermoFisher Scientific), lane 1—*Eco* RI, lane 2—*Hind* III, lane 3—*Bam* HI and lane 4—*Kpn* I, lane 5—uncut phage DNA. [The original gel images are presented in Supplementary File 1, Figures S1, S2 and S3].

The phages were further characterised by determining the restriction profile of the isolated DNA. All the extracted DNA had a molecular weight larger than 10 kb, and the digestion of the extracted DNA with the restriction enzymes (*Eco* RI, *Hind* III, *Bam* HI and *Kpn* I) indicated that all were double-stranded DNA viruses. When digested with restriction enzymes, the phages differed in their digestion profile. Phage ϕPh_SE03 was digested with both *Eco* RI and *Bam* HI, phage ϕPh_SD01 was sensitive to only *Hind* III digestion, and ϕPh_EC01 was digested with only *Bam* HI (Fig. 5).

#### Phage-mediated reduction of target bacteria

**Figure 6:**
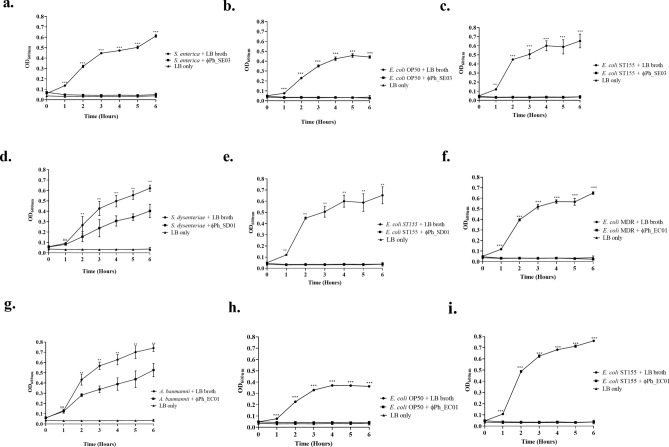
Bacteriolytic assay of all susceptible hosts treated with respective phages propagated in *E. coli* ST155 at a multiplicity of infection 10. Absorbance at 600 nm is measured every 1 h for 6 h. Phage ϕPh-SE01 treated (**a**) *S. enterica*, (**b**) *E. coli* OP50, (**c**) *E. coli* ST155, ϕPh_SD01 treated, (**d**) *S. dysenteriae*, (**e**) *E. coli* ST155 and ϕPh_EC01 treated, (**f**) *E. coli* MDR, (**g**) *A. baumannii*, (**h**) *E. coli* OP50 and (**i**) *E. coli* ST155. The error bar represents standard deviations three replicates from independent experiments, and a *p*-value ≤ 0.05 were considered significant.

Using a liquid medium assay, the host range and bacteriolytic potential of the polyvalent phages propagated in the ST155 were confirmed. Following treatment with the phages, all the alternative hosts and the production host showed a significant reduction in their cell growth compared to the control without phage treatment. After adding the phage, the cultures were lysed rapidly within 1 h, and the optical density was significantly reduced to blank levels of more than 90% until 6 h of treatment. As can be seen, the optical density of the control increased throughout incubation until the culture reached the stationary phase (Fig. 6). There was, however, a slight difference in the growth kinetics of *S. dysenteriae* and *A. baumannii* following phage treatment (Fig. 6d,g). Both bacteria had a lower optical density in the treatment, i.e., more than 30%, than in control, although their effect is less pronounced than on the other hosts. It may be attributed to the development of phage-resistant bacteria that has brought about a lesser reduction in these hosts.

### Characterisation of the production host *E. coli* ST155

#### Emergence of resistant mutants

The frequency at which bacteriophage insensitive mutants emerge when propagated in the production host *E. coli* ST155 was determined at an m.o.i. 10 at 37 °C. With respect to bacteriophage insensitive mutants (BIMs) frequency, there was no significant difference between 24 and 48 h in all three phages. On the other hand, both ϕPh_SE03 and ϕPh_SD01 showed a lower frequency when compared to ϕPh_EC01 (Table 3).

**Table 3:** Frequency of development of bacteriophage insensitive mutants of *E. coli* ST155 following incubation at 24 h and 48 h at an m.o.i. of 10 determined by double agar overlay method. SD represents standard deviations from three replicates from independent experiments.

Production host	Phage	BIMs frequency (CFU/mL)			
		24 h	SD	48 h	SD
*E. coli* ST155	ϕPh_SE03	3.29E−06	1.42E−06	4.66E−06	1.26E−06
	ϕPh_SD01	7.12E−06	1.26E−06	8.22E−06	8.22E−07
	ϕPh_EC01	3.45E−05	2.57E−05	4.60E−05	4.41E−05

#### Survival of *E. coli-infected* nematode

**Figure 7:**
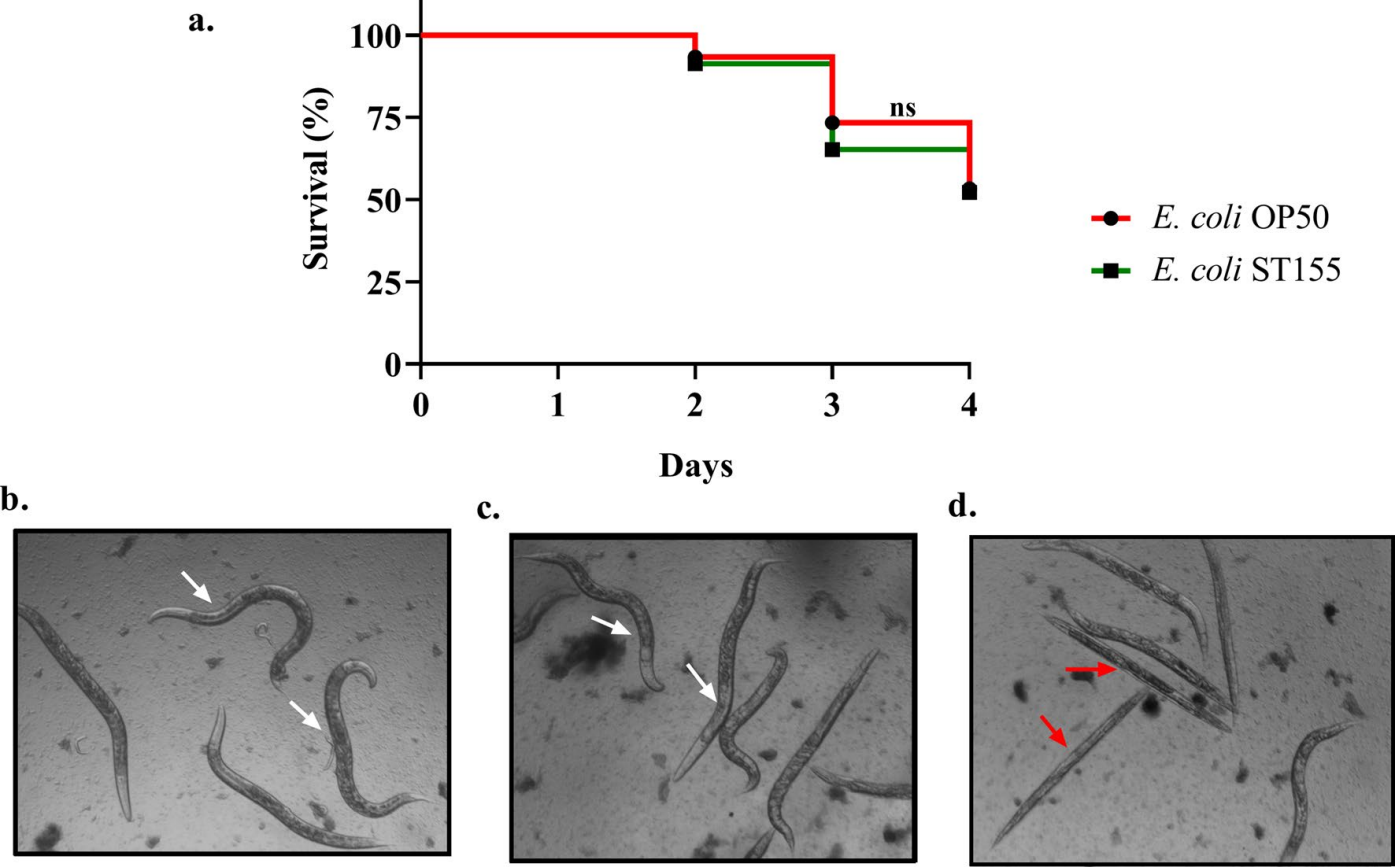
Survival assay of *C. elegans* after treatment with production host *E. coli* ST155. (**a**. Kaplan–Meyer curve shows the survival of *E. coli* ST155 monitored continuously for 4-days post-infection. *E. coli* OP50 was used as a negative control. (**b**) Photographic images showing gravid adults scored as live when fed with *E. coli* OP50 (white arrow). (**c**) Live gravid adult worms infected with *E. coli* ST155 (white arrow). (**d**) Worms scored as dead (red arrow). *P*-value ≥ 0.05 were considered non-significant from three independent experiments with replicates.

*Caenorhabditis elegans* has been used widely as a model organism for experimental bacterial infections, and 36% of its protein has human homologues making it a simple animal model for understanding molecular bacterial pathogenesis^17^. Worms were scored by the distinct differences between their live and dead appearance following infection. It was assumed that the worm's survival on OP50 would be a baseline for the healthy growth of the infected bacteria. The growth of the worms infected with *E. coli* ST155 was the same as that of OP50-fed worms, showing no significant influence on the survival of the worms after 4 days of infection (Fig. 7a). The live worms in the infected wells maintained the sinusoidal 'S' shape posture with an observable pharyngeal pumping and moved actively even after 4 days of exposure to *E. coli* ST155. They grew and became egg-laying, gravid adults (Fig. 7b,c). The worms that were considered dead are indicated in Fig. 7d. However, the results indicated that the production strain was non-pathogenic to the nematode.

#### Evolutionary relationship of *E. coli* ST155

**Figure 8:**
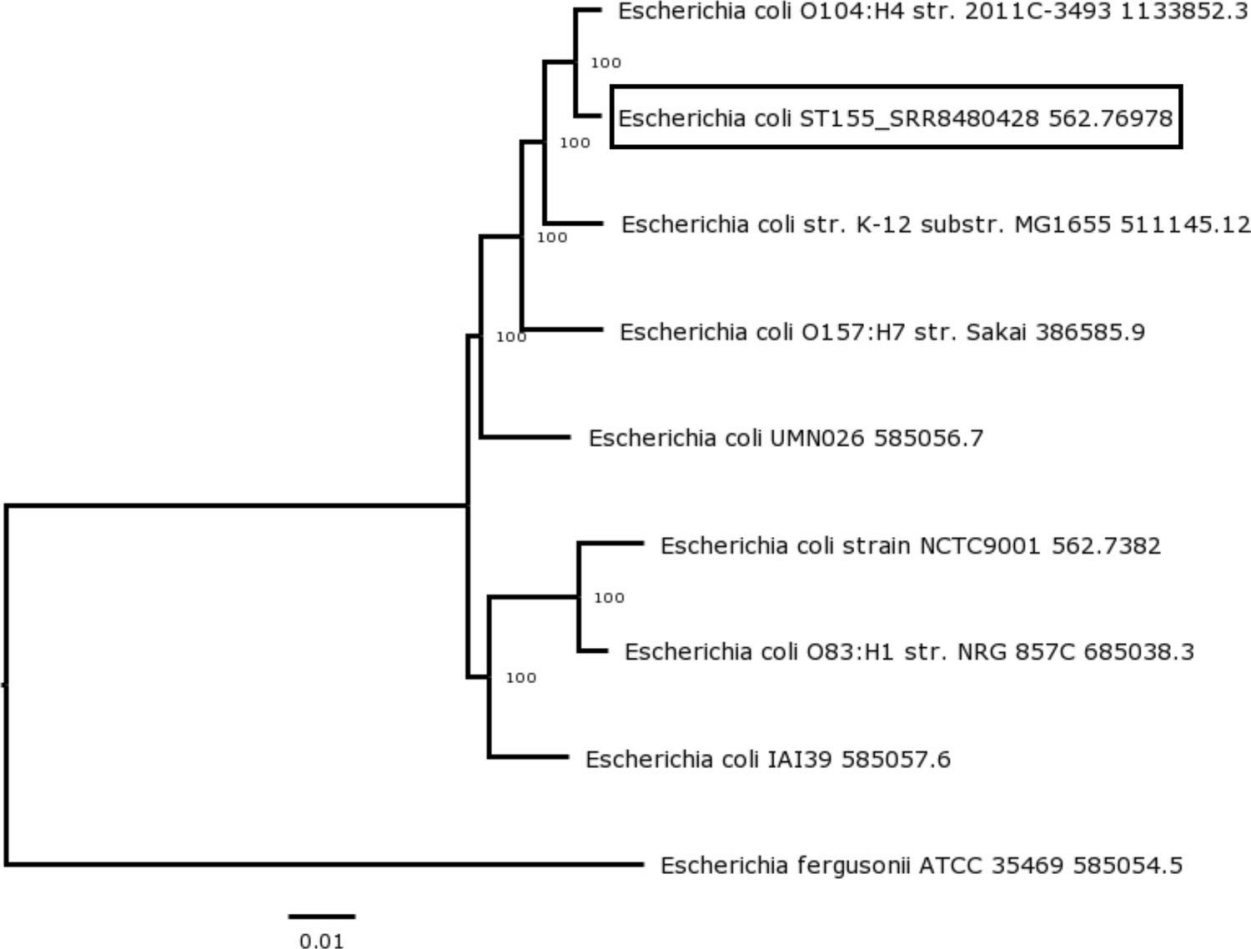
Analysis of *E. coli* ST155 genome with accession number RWJY00000000. Phylogenetic tree of *E. coli* ST155 shows the evolutionary relationship of the whole genome sequence of the isolate constructed using the Codon Tree Method that selects 500 single-copy genes (PATRIC PGFams). The branches are scaled in terms of the expected number of substitutions per site.

The phylogenetic tree of *E. coli* ST155 revealed that the host shares an evolutionary relationship with *E. coli* O104:H4, the strain reported being responsible for the spread of the German epidemic in 2011 (Fig. 8). The presence of phage-mediated Shiga toxins (*Stx*) produced by the strain of O104:H4 was responsible for the enterohemorrhagic nature of the German strain. However, a thorough analysis of the whole genome sequence of our host *E. coli* ST155 confirmed that the strain did not carry the *Stx* genes—*Stx1* and *Stx2*, rendering the strain a non-Shiga toxin-producing *E. coli*. This was further corroborated by validating the findings with the Centre for Genome Epidemiology (CGE) online bioinformatic resources (Supplementary File 2). Furthermore, no enterotoxigenic *E. coli* (ETEC) virulence factor genes involved in adherence (such as *cfaB*, *cofA*, *cooA*, *cs3*, *csbA*, *cseA*, *csfA*, *csnA*, *cssA*, *csvA*, *cswA*, *etpA*, *etpB*) or as toxins (such as *eltA*, *eltB*, *estIa*) were identified using the VirulenceFinder hosted by CGE.

#### Absence of antibiotic resistance and prophages in *E. coli* ST155

The antibiotic resistance profile indicates that ST155 is sensitive to almost all classes of tested antibiotics (Table 4). This observation was confirmed through two strategies. Firstly, the in vitro antibiotic sensitivity test yielded the above outcome which was secondly consolidated through validation with the ResFinder tool hosted by CGE as presented in Supplementary File 3. Additionally, induction with mitomycin clearly establishes the absence of prophages as it did not yield any visible phage lysis against *E. coli* ST155 as presented in Supplementary File 1: Figure S4.

**Table 4:** Antibiogram profile of *E. coli* ST155 against different classes of antibiotics.

Class of antibiotics							
β-lactam	Monobactam	Aminoglycoside	Fluroquinolone	Carbapenem	3G-Cephalosporin	Polymyxin	Sulphonamide
Ticarcillin	Aztreonam	Tobramycin	Levofloxacin	Meropenem	Ceftazidime	Colistin	Co-trimazole
S	S	S	S	S	S	S	S

Symbol S represents sensitivity.

#### Selective disinfection of *S. enterica* in wastewater

**Figure 9:**
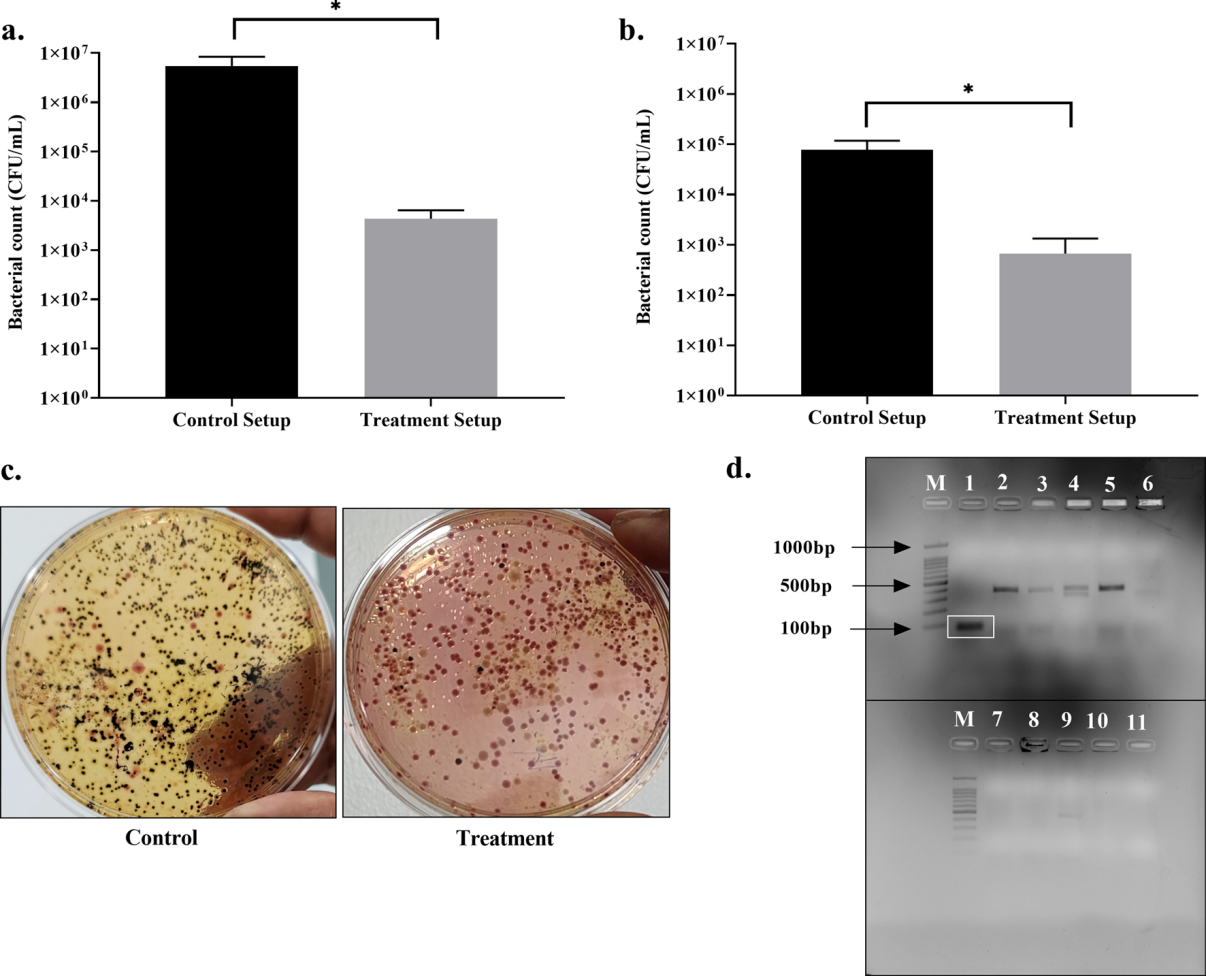
Bacteriophage-based treatment of *S. enterica-*augmented wastewater. (**a**,**b**) Reduction in *S. enterica* cells by 3-log in 24 h and 2-log in 96 h where phage ϕPh_SE03 was disseminated from a BAR-LBS employing *E. coli* ST155 as a phage production host into augmented wastewater. (**c**) Difference in the viable cell number of pigmented black colonies of *S. enterica* in SS agar between phage-treated wastewater and non-phage control. (**d**) Agarose gel showing colony PCR amplification of *invA* gene (77 bp) from morphologically different colonies picked from the SS agar plate of the treatment setup to confirm the specific reduction in *S. enterica* colonies and to differentiate from the non-target bacteria in wastewater. M—Marker 100 bp, lane 1—*S. enterica* (77 bp—highlighted in the white box), lane 2–10—non-targets, lane 11—non-template control. The error bar represents the standard deviation of three replicates from independent experiments and a *p*-value ≤ 0.05 were considered significant.

The effects of phages on the selective disinfection of *S. enterica* in 1 L of augmented wastewater were monitored and quantified by reducing viable cell numbers. Following 24 h of treatment with a BAR-LBS phage dissemination unit, the reduction in pigmented black colonies of *S. enterica* in the SS agar was 3-log (Fig. 9a,c), and the titre of amplified phage ϕPh_SE03 in the augmented wastewater was 1.9 × 10^8^ PFU/mL. At 96 h, the reduction was 2-log (Fig. 9b), and the subsequent phage titre was 1.8 × 10^8^ PFU/mL compared to the control containing augmented wastewater with non-phage BAR-LBS. The difference of 1-log between the 24 h and 96 h of the treatment can be attributed to the emergence of phage resistance among the bacterial pathogen, and the other plausible reason could be the lack of proper monitoring tools to capture the change in the overall microbiome dynamics of wastewater. Furthermore, colony PCR was used to confirm the selectivity of phages in treating wastewater to target specific pathogens. The pigmented black colonies of S. *enterica* showed an amplicon of 77 bp belonging to *invA* gene amplification following PCR while the rest of the other colonies did not amplify the gene, suggesting them to be the non-targets in wastewater (Fig. 9d).

## Discussion

The host range of a phage determines its impact on a microbial community, a narrow host range is a significant and frequently noted obstacle to phage-mediated interventions and biocontrol^39^. Phages are often produced in their isolation hosts, which might be pathogenic; hence the risk associated with handling pathogenic organisms^13,40^. Moreover, the crude phage lysate prepared from such pathogenic hosts contains dead and living cells, cell debris and large amounts of endotoxins and exotoxins^41^. Recently there has been a renewed interest in polyvalent phages with a broad host range, including inter-generic and inter-family infectivity^16^. The use of polyvalent phages presents two advantages upfront, namely, improved efficiency^35^ and the use of a single host for their production^13^. Polyvalent phages in any environmental application can improve treatment if more than one bacterial pathogen needs to be addressed, particularly in a diverse ecosystem like wastewater, which contains many harmful bacteria. Benign production hosts can serve in the safe production and dissemination of phages as liquid formulations, immobilized preparation or through broadcasting devices (BAR-LBS). The rationale to use *E. coli* ST155 as a production host in this study was based on the observation that it is sensitive to different polyvalent phages, thereby enabling simultaneous dissemination of these into wastewater through BAR-LBS. The ability of the strain to host polyvalent phages can overcome one of the limitations of phage application, i.e., phages, by nature, are known to be species-specific, hence having limited scope in disinfection, especially in environmental settings^31,32^. Nevertheless, polyvalency helps to target a wide range of bacteria belonging to a different genus, thereby improving the efficiency of the phage-based strategy^33^.

Non-pathogenicity is another ideal feature of a production host which was established through an in vivo survival assay in *C. elegans*. Upon infection with the host, the worms did not significantly affect their life cycle from development to maturation relative to the control fed with *E. coli* OP50. Although the phylogenetic analysis of the host indicated an evolutionary relationship to Shiga toxin-producing *E. coli,* a whole genome sequence analysis confirmed the absence of Shiga toxin genes (*Stx1* and *Stx2*). Moreover, we could not identify any ETEC virulence factor genes involved in adherence and production of toxins, which further gave us the confidence to use this as a production host. Thus, using this strain would reduce the risk of accidental administration of undesirable pathogens into wastewater during treatment and, to an extent, reduce the cost of large-scale production and purification of phages.

Absence of prophages and antibiotic sensitivity ensured that *E. coli* ST155 would not serve as a source of antibiotic resistance and virulence genes. Non-induction of virions in the production host by mitomycin is an indication of the absence of active prophages. Mitomycin through extensive DNA damage and consequential recruitment of SOS mechanisms suppresses the phage repressor, eventually inducing the prophages^18^. Reduction in the turbidity of *E. coli* ST155 on exposure to mitomycin over a 24 h time point at different concentrations (0.5, 1.3, and 3 µg/mL) indicates the action of mitomycin on cell viability due to DNA damage. However, the non-productive action of mitomycin in this scenario strongly indicates the absence of functional prophages in the genome of *E. coli* ST155. Sensitivity of production host to different classes of antibiotics also renders them ideal for environmental applications, as its inadvertent release into the wastewater for example would not result in the spread of resistance genes among other bacterial pathogens.

Three polyvalent phages, ϕPh_SE03, ϕPh_SD01 and ϕPh_EC01, with different tropism profiles were propagated in *E. coli* ST155. The phages were morphologically identified as tailed phages of the class *Caudoviricetes* by TEM analysis*.* Following propagation through the production host *E. coli* ST155, each phage showed a distinct host spectrum exhibiting inter-generic and inter-family infectivity, as reported in several other studies^16^. There was an overall difference in the growth characteristics and features, including the latent period, burst size, EOP and restriction digestion profile of each phage. The advantage of higher EOP in the production host in maintaining high titre, which is an ideal feature needs to be cautiously read along with the observation by Pingfen Yu et al*.* that the enrichment of polyvalent phages through a linear host can dramatically reduce the EOP on the alternative hosts^35^ which is observed in the current study as well. As expected, phage-resistant mutants developed against each phage when tested at an m.o.i. of 10 with a moderate frequency. There is a possibility that this is due to the difference in phage-host infection dynamics^36^.

Employing a non-pathogenic host in the production and dissemination of phages was reported to be a practical approach in biocontrol applications^13^. However, challenges are replete with regard to the encounter of phages with their respective hosts in which is crucial to phage-based application. Non-specific and non-productive binding of phages to non-targets, loss of phage titre due to entrapment in particulate organic material (thereby decreasing m.o.i.). Maintaining a high titre is a prerequisite in such a scenario. Continuous and prolonged dissemination through devices such as BAR-LBS could help mitigate these issues. BAR-LBS is a simple membrane-based prototype developed solely for broadcasting phages for wastewater biocontrol applications. It provides flexibility to produce phages and their cocktails depending on the demand in conjunction with a production host. In a small-scale application BAR-LBS with *E. coli* ST155 for the phage ϕPh_SE03 has reduced *S. enterica* in augmented wastewater by 3-log within 24 h and 2-log in 96 h. This non-linearity could be because of the development of phage resistance or the insufficiency in high-resolution monitoring of the complex microbiome dynamics in wastewater. Moreover, the selective disinfection of *S. enterica* via BAR-LBS was further corroborated by *invA* gene-based colony PCR.

In the experiment BAR-LBS is charged with an initial load of 3 × 10^10^ PFU/mL of ϕPh_SE03. But the titre is dynamic since the device also accommodates the production host (6 × 10^9^ CFU/mL) which results in infection and phage amplification. Passive diffusion from the BAR-LBS was previously reported to be 9.3 × 10^6^ PFU/mL/h^19^, hence the efficiency of transfer from the device would maintain sufficient titre in wastewater. Nevertheless, the reduction in titre that was observed during the disinfection (when compared to the phage titre in the BAR-LBS) could hence be due to sequestration of phages or exhaustion of targets over a period of time. The advantage of using such a continuous system is in maintaining an adequate phage-host ratio which in turn would ensure a productive infection of the target pathogen. More importantly, this would compensate for the decay of phages and the loss of phages through hetero aggregation^20^ by decoys such as sludge flocs, particulate matter and other non-targets through non-specific adsorption^38^.

In conclusion, this study proves a potential strategy for employing phages in isolation as an alternative disinfection agent for targeted control of problematic pathogens or could supplement some biocides and disinfectants to improve treatment efficiency. Moreover, host-mediated propagation of phages in conjunction with BAR-LBS would undoubtedly hasten the translation process.

## Materials and methods

### Bacterial and nematode culture conditions

All the bacterial strains used in the study were maintained in the Luria Bertani agar (LB agar, HiMedia, India) and cultured in LB broth (HiMedia, India), incubated at 37 °C overnight. Culture plates were stored at 4 °C throughout the study. The wild-type *Caenorhabditis elegans* strain N2 was maintained at 20 °C in nematode growth medium agar (Peptone—2.5 g/L, NaCl—2.9 g/L, Agar—17 g/L, CaCl_2_—1 mM, KH_2_PO_4_—25 mM, MgSO_4_—1 mM) supplemented with 1 mg/mL of cholesterol (Sigma-Aldrich, USA) and pre-seeded with live *E. coli* OP50 as described previously^21^.

### Isolation and propagation of phages

Three polyvalent phages were isolated from the domestic wastewater against different enterobacterial clinical strains: *S. enterica*, *S. dysenteriae*, and *E. coli* MDR*.* To isolate the phages, wastewater samples were collected from the effluent treatment plant (ETP) at the School of Biotechnology, Amrita Vishwa Vidyapeetham, Kerala, India. Wastewater of 22.5 mL was enriched with 2.5 mL of deca strength phage broth (Peptone—100 g/L, yeast extract—50 g/L, NaCl—25 g/L, K_2_HPO_4_—80 g/L), inoculated with the respective host bacterium of 0.5 mL (OD_600_ ~ 1.0), followed by incubation with shaking at 200 rpm at 37 °C overnight. The supernatant was collected after centrifugation at 7000×*g* for 15 min at 4 °C followed by 0.22 µm (Cole-Parmer, India) membrane filtration to achieve bacterial cell-free lysates. The phages were detected by spotting 10 µL of the lysate on the host bacterial lawn on LB agar using a spot assay. A single plaque from each phage lysate was then purified following three rounds of passages, and the titre was determined using the double agar overlay method^22^.

The phages were named ϕPh_SE03, ϕPh_SD01, and ϕPh_EC01, respectively against *S. enterica*, *S. dysenteriae* and *E. coli* MDR. All the phages were then propagated in *E. coli* ST155-the production host. The strain is an environmental isolate of wastewater reported previously by the authors^23^. Briefly, the phages were enriched by inoculating 25 mL of single strength phage broth with 0.5 mL of *E. coli* ST155 (OD_600_ ~ 0.8–1.0). The sample was then incubated by shaking at 200 rpm at 37 °C till the culture reaches an exponential growth phase followed by infection with 0.5 mL of respective phage lysate. As described previously, the lysate was prepared by filtering the sample. The titre was then determined using agar overlay assay. All the experiments were performed with phages propagated and maintained in the production host *E. coli* ST155.

### Host specificity

Bacterial strains used in this study are listed in Table 5. All the clinical strains including strains isolated from wastewater were identified by 16S rRNA gene ribotyping. The host range of the phages following propagation in the production host was determined by standard spot assay^24^. Briefly, each bacterial inoculum of an overnight culture was swabbed onto individual LB agar plates, and 10 µL of phage lysate (~ 10^8^ PFU/mL) was spotted and allowed to dry. The plates were inverted and incubated at 37 °C for 16–18 h. After incubation, the plates were evaluated for the zone of lysis/plaques to determine the lytic activity of the phages against the alternative hosts. Additionally, each phage lysate was serially diluted in SM buffer and 10 µL of the dilutions were spotted in triplicate on the lawn of *E. coli* ST155 and the plates were incubated overnight at 37 °C to confirm the presence of phages but not bacteriocins were responsible for the zone of lysis.

**Table 5:** List of bacteria used for phage host range study.

S. No	Bacteria	Source	Strain designation/accession number
**Gram-Negative Bacteria**			
1	*Salmonella enterica*	Clinical^a^	MW116733^2^
2	*Shigella dysenteriae*	Clinical^a^	ATCC 13313^1^
3	*E. coli*	Clinical^a^	MDR^1^
4	*E. coli*	Laboratory^b^	OP50^1^
5	*Klebsiella pneumoniae*	MTCC^c^	3384^2^
6	*Klebsiella quasipneumoniae*	Clinical^a^	–
7	*Klebsiella variicola*	Wastewater^d^	F2R9^1^
8	*Klebsiella aerogenes*	Laboratory^b^	–
9	*Serratia marcescens*	MTCC^c^	97^2^
10	*Vibrio cholerae*	Clinical^a^	ATCC 14035^1^
11	*Acinetobacter baumannii*	MTCC^c^	1425^2^
12	*Proteus vulgaris*	MTCC^c^	7299^2^
13	*Pseudomonas aeruginosa*	MTCC^c^	PAO1^2^
14	*Pseudomonas aeruginosa*	Clinical^a^	DSM 50071^1^
**Gram-Positive Bacteria**			
15	*Staphylococcus aureus*	Clinical^a^	MRSA^1^
16	*Lactobacillus fermentum*	Wastewater^d^	ASBT-2^1^

^a^Translational Health Science and Technology Institute, Delhi, India.

^b^Sanitation Biotechnology Lab, Amrita School of Biotechnology, Kerala, India.

^c^Microbial Type Culture Collection Center (MTCC), Chandigarh, India.

^d^Effluent Treatment Plant at Amrita School of Biotechnology, Kerala, India.

^1^Strain designation.

^2^Accession number.

### One-step growth curve

A one-step growth curve, as previously described^25^, determined the burst sizes and latent periods of phages. Briefly, 1 mL of *E. coli* ST155 (OD_600_ 0.2–0.3 i.e., ~ 10^8^ CFU/mL) was centrifuged at 13,000×*g* for 5 min. The pellet was washed thoroughly with the SM buffer (100 mM NaCl—5.8 g/L, 8 mM MgSO_4_·7H_2_O—2 g/L, 50 mM Tris–Cl—50 mL (pH 7.5), and 0.002% (w/v) gelatin solution), followed by adding 0.1 mL (~ 10^7^ PFU/mL) of phage to achieve an m.o.i. of 0.1 to 0.9 mL of the bacterial culture and incubated for 15 min at 37 °C to allow the adsorption of phage virions. Following incubation, the suspension was centrifuged at 13,000×*g* for 4 min at 28 °C. After the supernatant was removed, the pellet was resuspended in 10 mL of fresh LB broth and serially diluted until 10^−4^ dilutions. The tubes were kept for incubation at 37 °C at 200 rpm. During 90 min of incubation, 0.3 mL of the sample was withdrawn at different time points 0, 10, 20, 30, 40, 50, 60, 70, 80, and 90 min. The titre was determined immediately by a double agar overlay method. Burst size was calculated as the ratio of the average phages released in the baseline to the average bacteriophage count after the burst. The assay was performed independently with triplicates per experiment.

### EOP

Bacteriophages were tested for their EOP upon propagation through the production host as described previously in the literature^26^ to determine the productive infection of each phage against different sensitive bacteria. Briefly, the phage lysates were diluted in the SM buffer and 10 µL of each dilution was spotted in triplicates on the plates overlaid with the target bacteria. The plates were incubated overnight at 37 °C. Following incubation, EOP was estimated as the ratio of the average PFU with the alternative host (PFU_a_) to the average PFU with the production host (PFU_p_), i.e., PFU_a_/PFU_p_. The average EOP value for the phage-bacterium combination was classified as high, medium, and low, as indicated in the literature.

### Morphological characterisation

The high titre phage suspension (10 µL) was applied to the 200-mesh TEM grid for 1 min and stained negatively with a drop of 2% uranyl acetate for 1 min. The excess stain was removed using filter paper wicks and dried at room temperature. The grids were later analysed using FEI TECNAI G2 Spirit Bio Twin at 120 kV at the Central Instrumentation Facility, Indian Institute of Science Education and Research (IISER-TVM), Trivandrum, Kerala, India.

### Phage DNA isolation and restriction digestion

The phage nucleic acid was extracted using the buffered phenol–chloroform extraction protocol described previously^27^. Briefly, the PEG-precipitated (20% PEG 8000, 2.5 M NaCl) phage lysates of 1 mL (~ 10^9^–10^10^ PFU/mL) were pelleted and suspended in 500 µL of 5 mM MgSO_4._ Following the treatment with 1.25 µL each of DNase I (2000 U/µL, Ambion, Thermo-Fisher Scientific, USA) and RNase A (20 mg/mL, ThermoFisher Scientific, USA) at 37 °C for 1 h, the samples were further incubated with 1.25 µL of Proteinase K (20 mg/mL, Origin, India), 25 µL of 10% SDS, and 20 µL of 0.5 M EDTA (pH 8.0) at 60 °C for 1 h. The purification was followed by conventional buffered phenol: chloroform (1:1) extraction protocol. The extracted DNA was digested with restriction endonucleases: *Eco* RI, *Hind* III, *Bam* HI, and *Kpn* I (Takara, Japan). The buffers and digestion conditions were followed according to the manufacturer's instructions. After the enzymatic digestion, the DNA fragments were separated by electrophoresis in a 1% agarose (HiMedia, India) gel containing ethidium bromide (10 mg/mL, Sigma-Aldrich, USA).

### Bacteriolytic assay

The bactericidal activity of each phage was determined against each of the sensitive hosts in a time-dependent manner^28^. For each culture of 0.1 mL (OD_600_ 0.1 i.e., ~ 10^8^ CFU/mL), 0.1 mL of phage lysate (~ 10^9^ PFU/mL) were added at an m.o.i. of 10 into a sterile 96-well microtiter plate (Tarsons, Korea) in triplicate and were thoroughly mixed. The SM buffer was used as negative control while the LB-alone served as a blank. The microtiter plates were incubated following the addition of the samples at 37 °C at 200 rpm for a specified time interval of 0, 1, 2, 3, 4, 5, and 6 h. The reduction in bacterial growth/cell density in terms of its turbidity was measured at 600 nm in a microplate reader (BioTek, USA) to determine the lytic activity of the phages following propagation through the production host.

### BIMs frequency

The production host was evaluated for the frequency of development of BIMs against each of the three polyvalent phages^27^. Briefly, 100 µL of each phage (10^9^ PFU/mL) at an m.o.i. of 10 was incubated for 15 min at 37 °C with 100 µL of *E. coli* ST155 (10^8^ CFU/mL). Following incubation, samples were mixed with 4 mL of 0.7% soft agar and poured onto a 2% hard agar. Plates were incubated overnight at 37 °C, and all the surviving colonies were counted at 24 h and then at 48 h. The BIM frequency was calculated as the ratio between the surviving colonies and the initial number of bacteria incubated with the phage.

### *Caenorhabditis elegans* survival assay

For the survival assay using liquid media^21^, the N2 worms were initially synchronised by isolating the eggs from gravid adults via 30% sucrose solution, hatching the eggs overnight at 20 °C in 89% M9 buffer (minimal media—0.3% monobasic potassium phosphate, 0.5% sodium chloride, 0.6% sodium phosphate dibasic, 1 M magnesium sulphate) supplemented with 10% LB and 1% cholesterol without feed. Briefly, the overnight culture of *E. coli* ST155 was diluted to an OD_600_ 0.1–0.5 (~ 10^8^ CFU/mL), then washed free of LB broth thrice using M9 minimal media, and the final pellet was resuspended in M9 minimal media before infecting *C. elegans.* Following the egg hatching, ~ 20–30 L_1_-stage worms were transferred to the 96-well microtiter plate containing 200 µL of M9 buffer. The control wells were seeded with *E. coli* OP50 with an OD_600_ equivalent to the treatment and incubated at 20 °C. Over the next 4 days of incubation, the survival of the worms was scored manually. Worm survival was scored based on their active movement and pharyngeal pumping.

### Construction of phylogenetic tree of *E. coli* ST155

A codon tree based on up to 500 core genes was generated with PATRIC BRC^29,30^ to investigate the evolutionary relationship of *E. coli* ST155. The default is set for Codon Tree, which utilises amino acid and nucleotide sequences from up to 500 PATRIC's global Protein Families (PGFams). These are picked randomly to build an alignment and generate a tree based on the differences within those selected sequences. Both the protein (amino acid) and gene (nucleotide) sequences are used for each of the selected genes from the PGFams. Protein sequences are aligned using MUSCLE^31^, and the nucleotide coding gene sequences are aligned using the codon align function of BioPython^32^. A concatenated alignment of all proteins and nucleotides was converted to a phylip formatted file. Then a partitions file for RaxML^33^ was generated, describing the alignment in terms of the proteins and then the first, second and third codon positions. Support values were generated using 100 rounds of the "Rapid" bootstrapping option of RaxML.

### Validation of antibiotic resistance and virulence

Production host *E. coli* ST155 was tested to determine its antibiogram profile against different classes of antibiotics using the disk diffusion assay. Briefly, the strain was tested against antibiotics including, Ceftazidime (30 μg), Ticarcillin (75 μg), Levofloxacin (5 μg), Co-trimoxazole (25 μg), Tobramycin (10 μg), Aztreonam (30 μg), Meropenem (10 μg), and Colistin (10 μg). All the disks used in the assay were procured from HiMedia Laboratories, India. The disks were placed on the bacterial lawn 24 mm apart from the centre of each disk in a Muller-Hinton Agar. The zone of inhibition was measured and interpreted by CLSI recommendations^34^. The antibiogram profile was also validated using the CGE resources by submitting the assembled reads/contigs into the ResFinder tool^35–37^. Additionally, the presence of ETEC virulence genes was also checked with the VirulenceFinder tool^37–39^ hosted by CGE.

### Prophage induction

The strain *E. coli* ST155 was detected for the presence of prophages by inducing the culture with mitomycin C (10 mg, Merck, USA) as described previously in the literature with modifications^40^. Briefly, 200 µL bacterial culture in an early exponential phase (OD_600_ ~ 0.2) was chemically induced by adding mitomycin at a final concentration of 0 (i.e., without mitomycin), 0.5, 1.3 or 3 µg/mL. LB broth (200 µL) alone was used as a blank. The microtiter plate was incubated at 37 °C and the bacterial growth was followed for 0, 1.5, 3 and 8 h; the final readings were obtained at 24 h. To determine the presence of prophages, the cells treated with each concentration of mitomycin were collected after 24 h and were centrifuged at 7000×*g* for 2 min, the bacterial cell-free lysates were then subjected to spot assay against *E. coli* ST155.

### Disinfection of augmented wastewater

**Figure 10:**
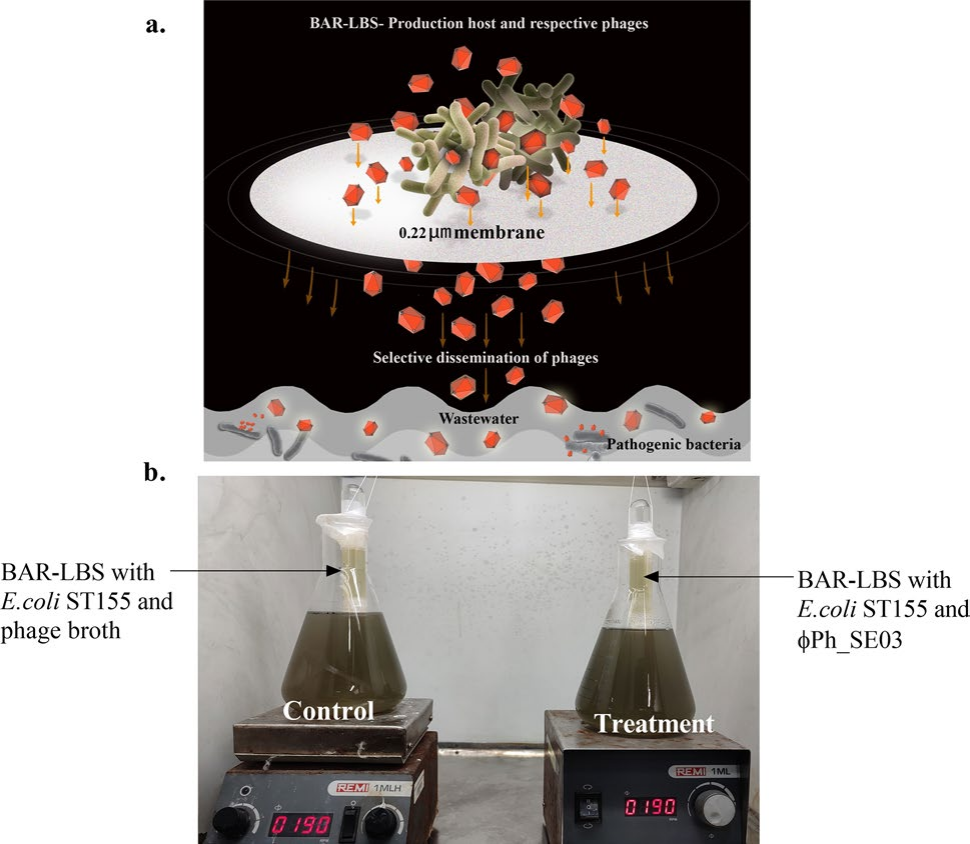
BAR-LBS employed for treating 1 L of *S. enterica* augmented wastewater. (**a**) Concept diagram of BAR-LBS; the device includes a contraption that opens up to wastewater through a 0.22 µm membrane. Production host on infection continually produces phages that are selectively (without bacteria) disseminated into wastewater across the membrane. (**b**) BAR-LBS experimental prototype (in control and treatment setup) introduced in wastewater.

The disinfection potential of phages was determined in wastewater augmented with an inoculum of *S. enterica* (10^5^ CFU/mL). BAR-LBS, whose fabrication is described in the literature, was charged with 6 × 10^9^ CFU/mL of *E. coli* ST155 infected with 3 × 10^10^ PFU/mL of phage ϕPh_SE03, with both constituting a volume of 40 mL. On the other hand, the control of BAR-LBS consisted of *S. enterica* and phage broth as a non-phage control. Each BAR-LBS was suspended into the respective control and treatment setups containing *S. enterica* augmented wastewater of 1 L (Fig. 10b). The passive and selective diffusion of phages through the membrane and the subsequent disinfection of the target pathogen was determined (Fig. 10a). Briefly, a sample of 2 mL was collected at 24 h and 96 h and subsequently plated in *Salmonella-Shigella* agar (SS agar, HiMedia, India). Simultaneously, the increase in the titre of phage ϕPh_SE03 was monitored.

### Colony PCR-based monitoring

The reduction of *S. enterica* following phage treatment was confirmed by performing colony PCR using the *invA* gene specific to *S. enterica*^41^. The colonies resultant from the treatment setup with different morphologies were randomly chosen and suspended in 50 µL of nuclease-free water (Fisher Scientific, USA). The cells were then disrupted by heating at 95 °C for 30 min. The PCR reaction of 12 µL was carried out with 1 µL of the cell lysate as the DNA template, 0.2 µL each of forward (5′- TCGGGCAATTCGTTATTGG-3′) and reverse oligos (5′- GATAAACTGGACCACGGTGACA-3′), 6 µL of 2X master mix (GoTaq Green, Promega) and 4.8 µL of nuclease-free water. The program consists of an initial 95 °C heating step for 10 min followed by 34 cycles of 95 °C for 15 s, 59 °C for 30 min, 72 °C for 30 s, and followed by a final extension of 7 min. The amplicon was separated in 2% agarose gel for visualisation.

### Statistical analysis

GraphPad Prism 8.0 (GraphPad Sofware Inc., San Diego, CA) was employed to perform all the statistical analyses. The data obtained from the bacteriolytic assays were analysed using the Two-way ANOVA of Variance followed by Tukey’s test, the disinfection experiment in wastewater was analysed with an unpaired, two-tailed t-test, and the survival assay was analysed using Log-rank (Mantel-Cox) test. The significance level was considered at *p*-value ≤ 0.05, and the data were expressed in mean values ± standard deviation.

## Supplementary Information

Supplementary Information 1.

Supplementary Information 2.

Supplementary Information 3.

## Data Availability

The whole genome sequence of *E. coli* ST155 was deposited at GenBank under the accession number https://www.ncbi.nlm.nih.gov/nuccore/RWJY00000000 (BioProject number PRJNA509104) with SRA accession number https://www.ncbi.nlm.nih.gov/sra/SRR8480428.
