# Supplementary material for: Escherichia coli ST155 as a production-host of three different polyvalent phages and their characterisation with a prospect for wastewater disinfection
Source: Sci Rep. 2022 Nov 12;12:19406. doi: 10.1038/s41598-022-24134-4 (PMC9653416; doi:10.1038/s41598-022-24134-4)
Supplement: Supplementary file 1 — Supplementary Information 1. [file 41598_2022_24134_MOESM1_ESM.docx]

***Escherichia coli* ST155 as a production-host of three different polyvalent phages and their characterisation with a prospect for wastewater disinfection**

Amrita Salim^1^, Ajith Madhavan^1,*^, Suja Subhash^1^, Megha Prasad^1^, Bipin G. Nair^1^, Sanjay Pal^1,*^

^1^ School of Biotechnology, Amrita Vishwa Vidyapeetham, Kerala – 690525, India

^*^Dr Sanjay Pal, [sanjaypal@am.amrita.edu](mailto:sanjaypal@am.amrita.edu)

*Dr Ajith Madhavan, [ajithm@am.amrita.edu](mailto:ajithm@am.amrita.edu)


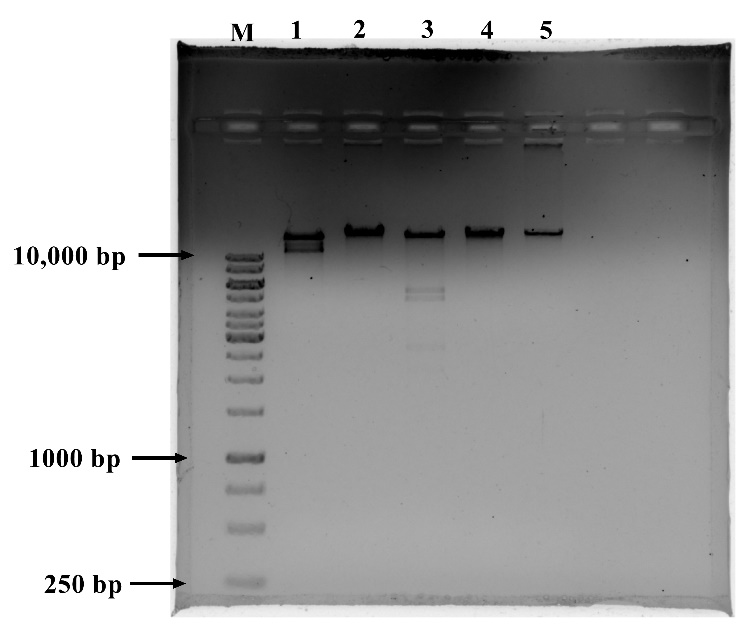


Figure S1: Restriction enzymes digested phage DNA of ϕPh_SE03 electrophoresed on a 1% agarose gel stained with ethidium bromide. M – Marker 1kb (GeneRuler, ThermoFisher Scientific), lane 1- *Eco* RI, lane 2- *Hind* III, lane 3- *Bam* HI and lane 4- *Kpn* I, lane 5- uncut phage DNA.


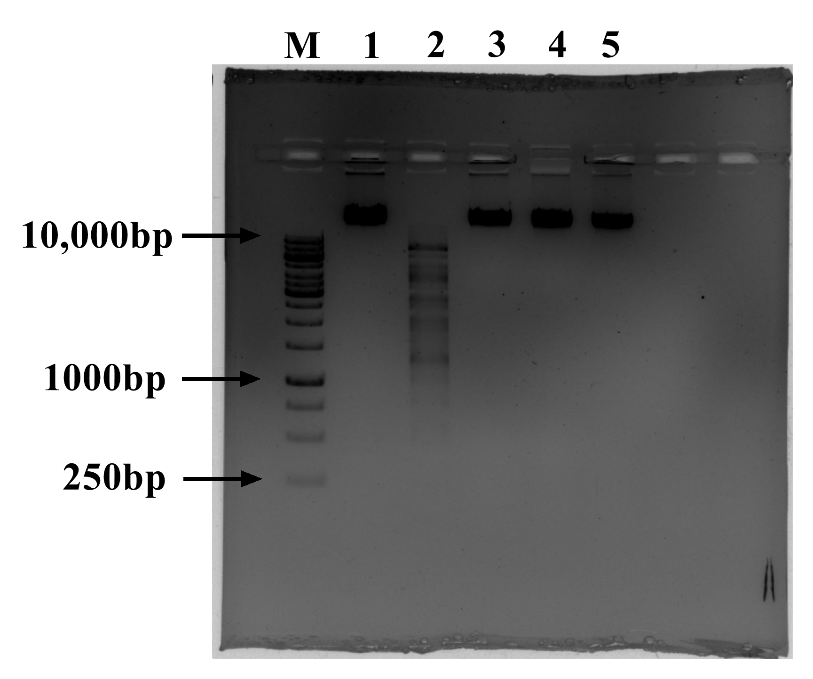


Figure S2: Restriction enzymes digested phage DNA of ϕPh_SD01 electrophoresed on a 1% agarose gel stained with ethidium bromide. M – Marker 1kb (GeneRuler, ThermoFisher Scientific), lane 1- *Eco* RI, lane 2- *Hind* III, lane 3- *Bam* HI and lane 4- *Kpn* I, lane 5- uncut phage DNA.


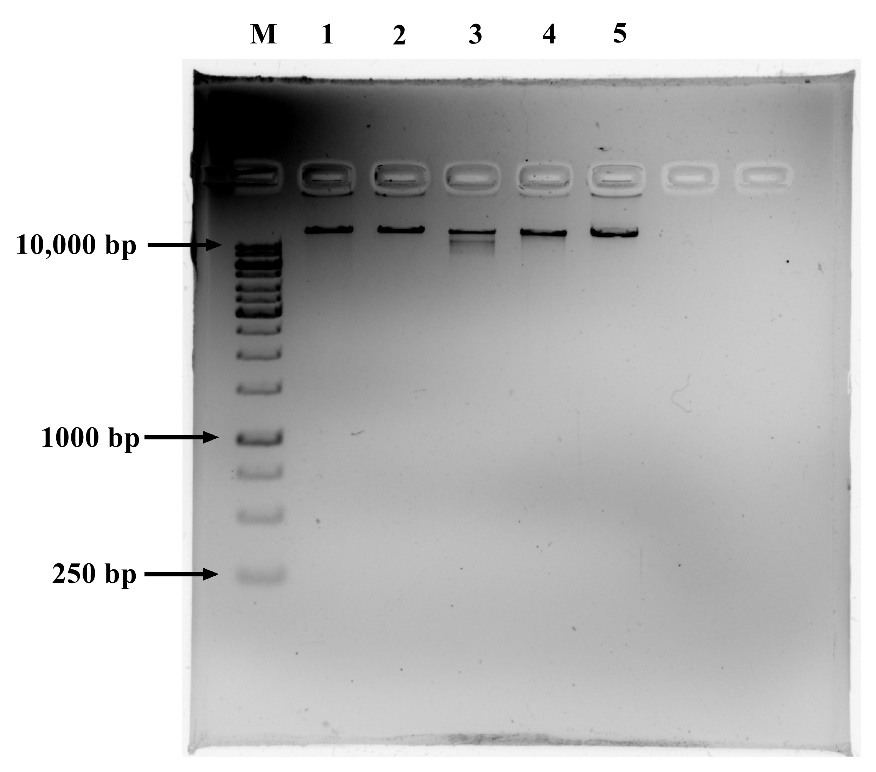


Figure S3: Restriction enzymes digested phage DNA of ϕPh_EC01 electrophoresed on a 1% agarose gel stained with ethidium bromide. M – Marker 1kb (GeneRuler, ThermoFisher Scientific), lane 1- *Eco* RI, lane 2- *Hind* III, lane 3- *Bam* HI and lane 4- *Kpn* I, lane 5- uncut phage DNA.


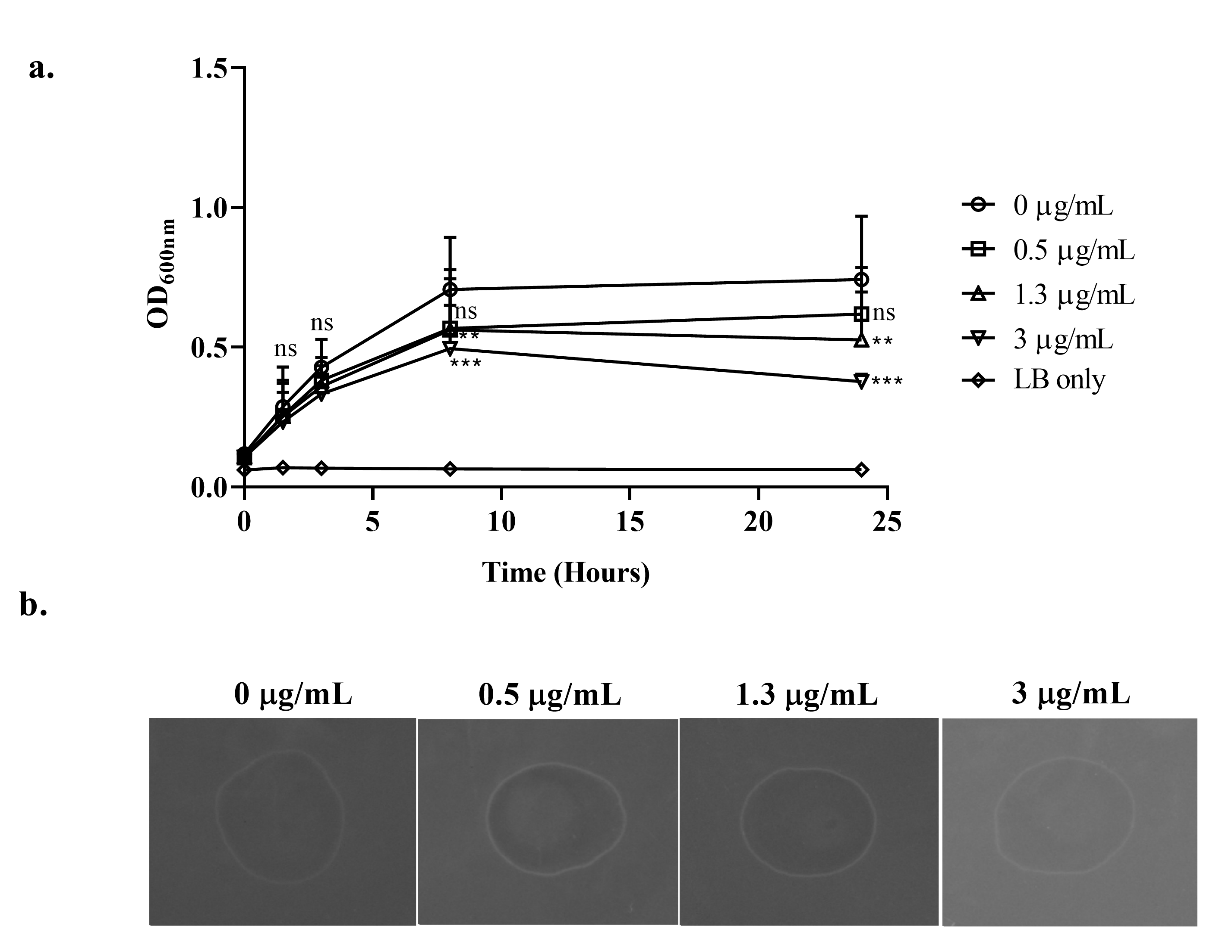


Figure S4: Induction of prophages in *E. coli* ST155 a. Mitomycin C induction of log-phase culture of *E. coli* ST155 at different concentrations for a time period of 24 h shows a reduction in the turbidity of the culture. b. Spot assay of the cell-free lysate collected following mitomycin induction from different concentrations shows no infective lysis/plaques against *E. coli* ST155 indicating the absence of functional prophages. SD represents the standard deviations of five replicates from independent experiments.


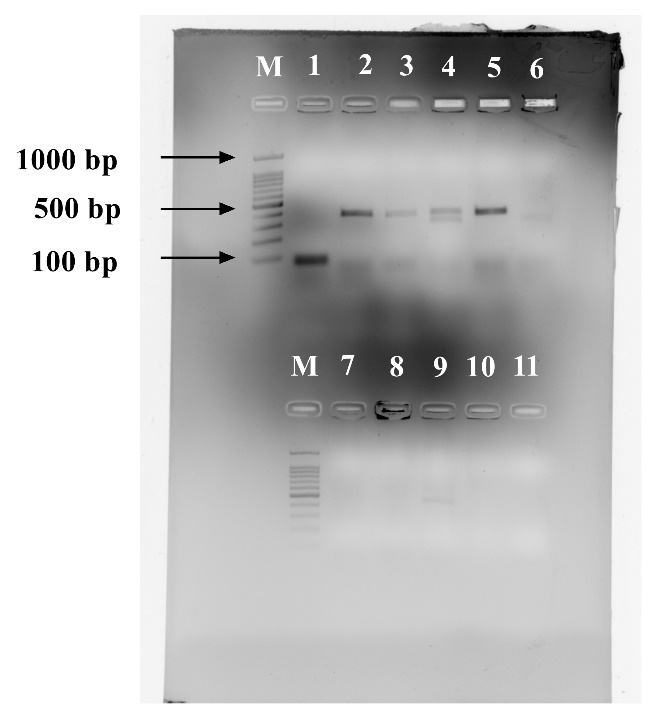


Figure S5: Agarose gel showing colony PCR amplification of *invA* gene (77 bp) from morphologically different colonies picked from the SS agar plate of the treatment setup to confirm the specific reduction in *S. enterica* colonies and to differentiate from the non-target bacteria in wastewater. M- Marker 100 bp, lane 1 - *S. enterica* (77 bp- highlighted in the white box), lane 2-10 - non-targets, lane 11 - non-template control.
